# A Genome-Wide Modeling and Characterization Study of Pleckstrin Homology Domains in *Chlamydomonas reinhardtii*

**DOI:** 10.3390/plants14172607

**Published:** 2025-08-22

**Authors:** Münevver Aksoy, Marina Krupitskaya, Shaneen M. Singh

**Affiliations:** 1 Department of Agricultural Biotechnology, Akdeniz University, Antalya 07070, Türkiye; 2 Department of Biology, Brooklyn College, City University of New York, Brooklyn, NY 11210, USA; marina.krupitskaya@brooklyn.cuny.edu

**Keywords:** PH domain, *Chlamydomonas reinhardtii*, protein structure, homology modeling, phosphoinositide

## Abstract

The function of pleckstrin homology (PH) domains is to recognize and bind to specific phosphoinositides in the membranes as part of diverse cellular signaling processes. The structure of some PH domains has been solved by X-ray crystallography, but structures of many PH domains remain to be elucidated. In green alga *Chlamydomonas reinhardtii*, none of the PH domains have been crystallized or characterized. The goal of our study was to model and characterize in detail the structures of all eleven of the PH domains identified in *C. reinhardtii*. Our computational strategy of integrating the information available on sequence, structure, and function with modeling and biophysical characterization has uncovered new biological predictions for these proteins. These predictions can be validated by future rationally designed experimental studies as an extension of this work. Our results suggest that nine of the eleven *C. reinhardtii* PH domains show the classical electrostatic polarization of PH domains with a positively charged binding pocket and negatively charged opposing end. Our docking results predict only two PH domains bind specifically to a particular phosphoinositide, while all the other nine PH domains may be able to bind various inositol phospholipids. The lack of preference for a specific phosphoinositide headgroup implies that the positive charge in the binding pocket of the PH domains may be crucial in driving the interaction with the negatively charged phosphoinositides in a non-specific or promiscuous manner. We identified putative homologs of Dynamin GTPase, calcium/calmodulin-dependent kinase, Arf GAP, Rhythm of Chloroplast 23 (ROC23), and oxysterol binding proteins in *C. reinhardtii* that contain PH domains. In addition, we identified two PH domain-containing proteins that may play a role in the mating process and others that may be important for signaling under phosphate deficiency.

## 1. Introduction

Pleckstrin homology (PH) domains are small protein domains of around 130 amino acids that are found in proteins whose functions involve vesicular trafficking, signal transduction, cytoskeleton rearrangement, and various other cellular processes. Structural data suggest that the main function of PH domains is to recognize and bind to specific phosphoinositides in the membranes as part of these cellular processes [1,2]. However, they also bind other targets such as proteins [3,4].

The PH domain fold is characterized by a beta sandwich consisting of seven β-strands that is capped by an alpha-helix present at the C terminus. PH domains are lipid binding modules that establish association of peripheral membrane proteins with the membrane surface. Interaction of peripheral membrane proteins with the cell membrane is essential for many cellular signaling events. PH domains are best known for their ability to target cellular membranes through specific or non-specific interactions with phosphoinositide lipids [5]. The membrane binding function of many PH domains can be attributed to their electrostatically polarized nature. In this classical or typical electrostatic surface profile, the variable loops forming the membrane binding pocket of the domain is positively charged while the opposite surface of the PH domain is negatively charged [5,6]. Further, some PH domains can be classified as high-affinity, high-specificity binders recognizing a specific phosphoinositide, but others show low-affinity, non-specific or promiscuous binding to various phosphoinositides [5,7]. The PH domain binds to acidic phospholipids in the cytosolic side of the plasma membrane using the basic pocket in both types of binding modes. However, some PH domains can exhibit atypical or no electrostatic polarization. In these cases, a different region of the PH domain is involved in membrane targeting, or surface electrostatics does not play a key role [8,9]. Interestingly, there is low sequence identity among PH domains [5,6,7], yet they maintain the overall core structural fold and, in most cases, their canonical function of membrane targeting.

Proteins with diverse functions, such as oxysterol binding proteins [10], ArfGAPs [11,12], and dynamin GTPases [13,14] are known to have PH domains. PH domain-containing proteins are associated with various diseases and are potential targets for disease treatment [15]. A proteomic study performed in dairy cows infected with *Staphylococcus aureus* shows that there was an increase in PH domain-containing proteins in infected animals [16]. Targeting the PH domain of Akt with a natural compound called swertiamarin has shown promising results for the treatment of inflammation. The results show that swertiamarin’s anti-inflammatory activity stems from its ability to target the Akt PH domain and inhibit downstream inflammatory molecules [17]. PH domain-containing proteins are reported to be also involved in plant disease responses [18]. It has been shown that an *Arabidopsis* PH domain-containing protein, Enhanced Disease Resistance 2 (EDR2), is a negative regulator of cell death and mutants of *EDR2* have improved resistance against fungal pathogens [18,19]. Another study showed that mutants of *Arabidopsis* SWAP70, another PH domain-containing protein, have increased resistance against bacterial pathogens compared with wild-type plants [20]. These studies are a few examples of many studies that highlight the important functional roles mediated by PH domain-containing proteins in many different organisms.

The green alga *Chlamydomonas reinhardtii* has been used as a model organism in basic research and biotechnology to study many cellular processes, such as photosynthesis and mating [21,22,23,24]. However, PH domain-containing proteins and their constituent PH domains have not been investigated in this important model organism, highlighting a big gap in knowledge. Identifying and characterizing PH domains in *C. reinhardtii* can benefit the broader algal research community, e.g., by advancing the understanding of membrane-associated signaling or vesicle trafficking in algae. In addition, these genome-wide analyses of PH domains will help to understand the function of novel genes encoding PH domains that have previously not been associated with any functional annotation. The objective of this study was to identify, model, and characterize the three-dimensional structures of all PH domains found in the *C. reinhardtii* genome. This is the first study that provides valuable foundational information for understanding the role of PH domains in this important model organism.

## 2. Results

### 2.1. Protein Sequence Analysis and Cellular Localization Predictions

**Figure 1:**
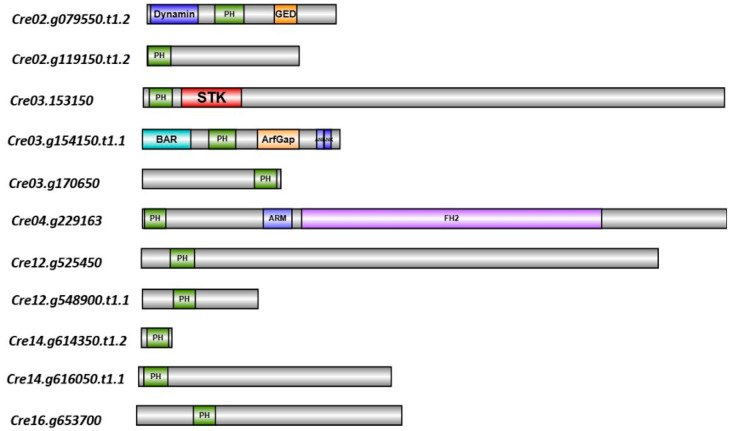
Domain architecture of the PH domain-containing proteins. PH domains are colored green. Dynamin—Dynamin GTPase domain; GED—GTPase Effector Domain; STK—Serine threonine kinase domain; BAR—BAR domain; ArfGap—GTPase activating protein for Arf domain; ANK—Ankyrin repeat; ARM—Armadillo repeat; FH2—Formin homology2 domain.

We identified eleven gene models of PH domain proteins using a comprehensive search strategy (detailed in the methods, Section 4.1) and retrieved them from Phytozome [25] and AlgaePath [26] databases. Domain architecture analyses confirmed that all of the gene models housed a PH domain (Figure 1).

Phytozome annotations, predictions of cellular localization, isoelectric point (pI), and molecular weight of these sequences are detailed in Table 1. According to Phytozome annotations, three of these genes (Cre02.g119150.t1.2, Cre03.g170650, Cre14.g614350.t1.2) have no known functions. Although their specific function is also not known, Cre12.g525450.t1.1 and Cre14.g616050.t1.1 have mating activation-specific expression, suggesting these two proteins might have membrane-associated functions, possibly during gamete fusion. Other proteins are annotated as dynamin GTPase, calcium/calmodulin-dependent kinase, Arf GAP, ROC23, and formin-like and oxysterol binding proteins. Our protein localization predictions suggest that most of the proteins are likely localized in the cytosol, as expected, considering the roles of PH domains in membrane and cellular trafficking-related functions. However, three of them are predicted to localize in mitochondria by the PredAlgo tool, athough the DeepLoc tool predicted their localization as cytosolic.

### 2.2. Orthologs and Phylogenetic Analysis

A phylogenetic analysis of the 11 sequences was performed along with their identified *V. carterii* and *A. thaliana* orthologs. We also perfomed BLAST 2.17.0+ searches to find if any of the PH domain proteins had orthologs in other species and to provide clues as to putative functional annotation. Interestingly, five of the proteins (Cre02.g119150, Cre03.g170650, Cre04.g229163, Cre12.g525450, Cre14.g616050) had orthologs only in algae. Additionally, out of these five proteins, four have no known or predicted functions. However, Cre04.g229163 is annotated as a formin-like protein in the Phytozome database (Table 1). Cre14.g616050.t1.1 had algal orthologs, but did not have any orthologs in *V. carterii* and *A. thaliana*.

**Figure 2:**
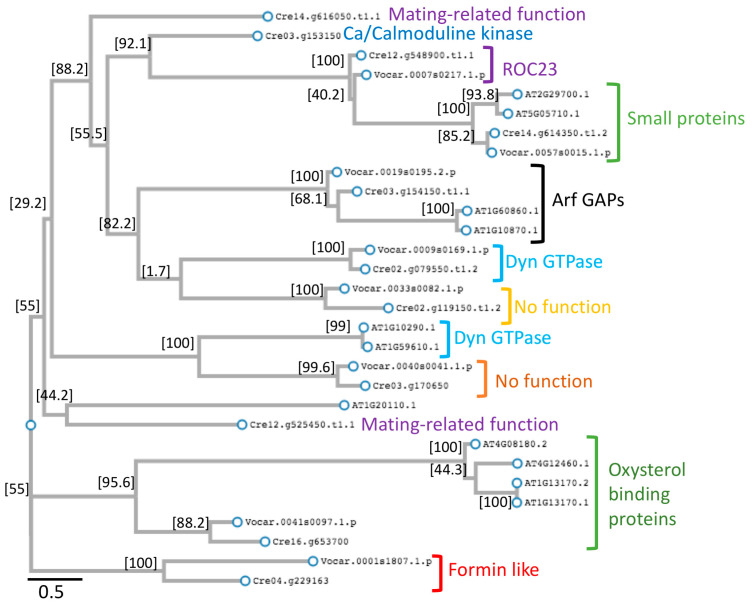
Phylogenetic analysis of *C. reinhardtii* PH domain-containing proteins and orthologs in *V. carterii* and *A. thaliana*. Only algal orthologs for Cre02.g119150, Cre03.g170650, Cre04.g229163, Cre12.g525450, and Cre14.g616050 were detected using BLAST similarity searches. Numbers at the nodes represent local support values with the Shimodaira–Hasegawa test.

Except for dynamin GTPases, all the other proteins clustered with their orthologs. While the putative *C. reinhardtii* (Cre02.g079550.t1.2) and *V. carterii* dynamin GTPases clustered together, *A. thaliana* dynamin GTPases were clustered separately (Figure 2). Although the putative Ca/calmodulin kinase (Cre03.g153150) had orthologs, our domain search did not find any PH domain in these orthologs; therefore, they were not included in our phylogeny analysis. The *A. thaliana* ortholog (AT1G20110) of the ROC23-like protein (Cre12.g548900) did not have a PH domain, but had a FYVE domain (an alternate membrane binding domain fold) and was included in the phylogeny analysis. Interestingly, this ortholog did not cluster with Cre12.g548900, and instead clustered with Cre12.g525450.

We found orthologs of the small *C. reinhardtii* protein (Cre14.g614350.t1.2) in both *V. carterii* and *A. thaliana*, suggesting that these small proteins seem to be constituted by a PH domain only, are also present in other organisms and might have conserved functions. These orthologs clustered together, suggesting they may have similar functions, although no known functions were found in the databases.

Cre12.g525450 has orthologs only in some algal species, such *Volvox africanus*, but we could not find any orthologs in *V. carterii*. This unanticipated finding may be due to limitations in ortholog detection in the *V. carterii* genome. We suggest this may be due to a limitation in detection since the *V. carterii* genome has not yet been fully completed. While it seems that this protein is found in algal lineages, other lineages such as higher plants do not have this protein. It is interesting that this protein may have a function in the mating process.

It should be noted that it is difficult to make a conclusion if the results reflect lineage-specific innovations or potential limitations on ortholog detection for some of the proteins, because of unfinished genome sequencing of some species or gaps in the genome annotation status.

### 2.3. Homology Models

**Figure 3:**
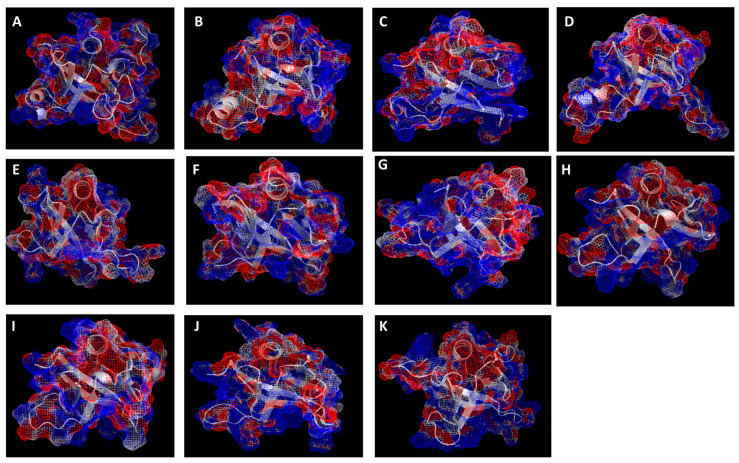
Mesh electrostatic profiles of PH domains. PH domain C-alpha trace shown as a white ribbon surrounded by mesh electrostatic representation visualized in Pymol. Blue—positive; red—negative (−4 to +4 kT/e). (**A**) Cre02.g079550, (**B**) Cre02.g119150, (**C**) Cre03.g153150, (**D**) Cre03.g154150, (**E**) Cre03.g170650, (**F**) Cre04.g229163, (**G**) Cre12.g525450, (**H**) Cre12.g548900, (**I**) Cre14.g614350, (**J**) Cre14.g616050, (**K**) Cre16.g653700.

Structure validation was performed for each model generated and the structure with the best validation score was chosen as the top-ranked model. The fold and electrostatic profiles of the best models are shown in Figure 3. Our top models were either comparable or better than the models generated by Alphafold (Supplementary Table S1). All the models have the canonical PH fold, which is characterized by a beta sandwich comprising seven β-strands capped by an alpha-helix present at the C terminus. The canonical binding site on the PH domain is formed by three variable loops at the open end of the β barrel, and the typical PH domain has strong positive electrostatic potential around the binding site. As can be seen in Figure 3, nine of the eleven models have positive charge (blue mesh) around this region, implicating it as the potential phosphoinositide binding site. Some of the PH domains instead showed a non-canonical electrostatic profile where the prominent positive potential is located at a non-canonical pocket.

### 2.4. Docking Analysis for Phosphoinositide Binding

**Figure 4:**
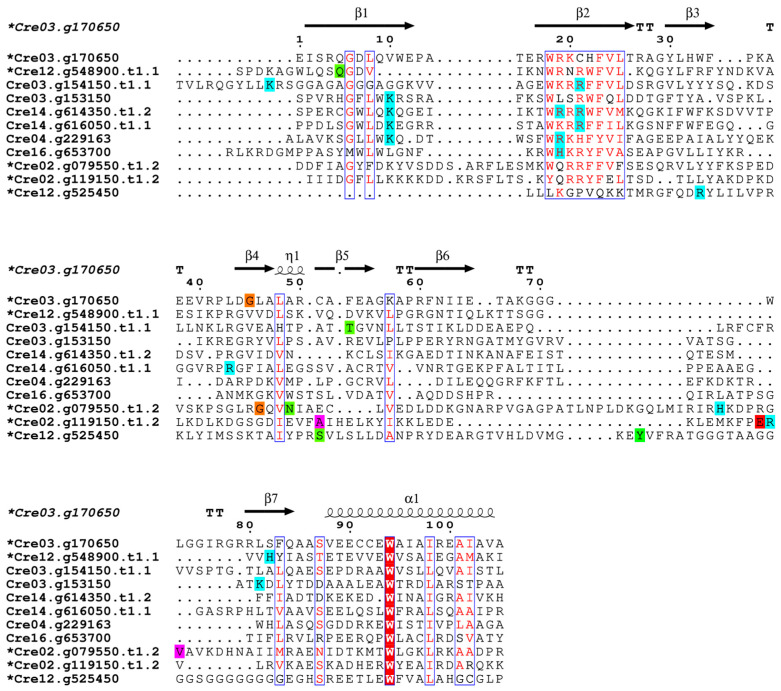
Structure-based sequence alignment of the *C. reinhardtii* PH domains showing PI-interacting residues based on docking analysis. The multiple sequence alignment (MSA) was created using Superpose and visualized in Espript. The MSA shows the highest-scoring docking scenario for each sequence. Identical residues are highlighted in red with white font, while regions of conservation are boxed in blue and similar residues are shown in red. The interacting residues identified based on docking analysis are highlighted based on physiochemical properties: positively charged—cyan, negatively charged—maroon, hydrophobic—magenta, aromatic—forest green, polar—citron green, glycine—orange. The sequences displaying binding in non-canonical pockets are marked with an asterisk next to the sequence name. Secondary structure elements of the modeled PH domain are shown at the top: β T, ⍺, and η represent β-strand, β-turn, alpha helix, and 310 helix, respectively.

PH domains are specific to one phosphoinositide or can bind various phosphoinositides [27,28]. Each PH domain 3D model was docked with a panel of seven phosphoinositides. The results of this analysis are summarized in Table 2 and Figure 4, and the detailed results are presented in Supplementary Figure S1. According to the docking results, Cre02.g079550.t1.2 and Cre03.g153150 exclusively bind I4P and I3P, respectively. All the other PH domains bind more than one PI species (Table 2).

Typically PH domains form the binding pocket with a major contribution from the β1 and β2 strands and the loop connecting the two strands. This is predicted for six of the *C. reinhardtii* PH domains with conserved basic residues involved in the binding. Interestingly, the other PH domains are predicted to bind to PIs using non-canonical binding pockets and residues (Figure 4).

To test the identified interface residues in the PH domains, we mutated the key binding residues for the highest-scoring phosphoinositide interaction for each of the 11 *C. reinhardtii* PH domains and assessed the impact of the mutations on the predicted interaction scenarios (Supplementary Table S2). As expected, for most interaction scenarios either no binding in the binding pocket was predicted or there were a reduced number of hydrogen bonds stabilizing the interaction. Interestingly, two of the non-canonical binders showed an increased number of hydrogen bonds when the key residues were mutated. The reason for this unexpected result is that the mutations made the non-canonical binding pocket less favorable and the canonical binding pocket offered a better binding pocket for these two PH domains.

## 3. Discussion

We identified eleven PH domain-containing proteins in the genome of the model alga *C. reinhardtii.* However, we did not find any functional studies associated with any of these genes in the literature. All eleven PH domains strongly suggest that they have the typical PH domain fold (Figure 3). The overall surface electrostatic profiles of the modeled PH domains indicate that for the most part, they show canonical polarization of charge and a typical binding pocket, as in other known PH domains [29]. Docking results show that apart from two *C. reinhardtii* PH domains, all the other PH domains bind various phosphoinositides, suggesting a promiscuous binding mode where non-specific electrostatics may be the predominant driving mechanism for membrane recognition, similar to the PH domains in the yeast genome [7,9].

PH domains are also known to bind protein targets such as the beta–gamma subunits of G-proteins [30], Protein kinase C [31], and small GTPases like ARF [32], and play a role in regulation of signaling via their protein partners. Two recent studies also show that PH domains may be involved in binding to protein targets in processes involving DNA repair. These studies shed light into how the TFIIH complex that is involved in nucleotide excision repair is recruited to DNA. According to these studies, the PH domain of the Tfb1 and p62 subunits of the TFIIH complex in mammalian and yeast cells, respectively, are involved in interacting with protein partners and mediating the recruitment of TFIIH to DNA [3,4]. None of the PH domains we analyzed in this study are predicted to be involved in DNA repair, but some have no known functions (Cre02.g119150.t1.2, Cre03.g170650, Cre12.g525450.t1.1, Cre14.g614350.t1.2, Cre14.g616050.t1.1). Using various protein–protein interaction methods, such as yeast-two hybrid, possible protein binding partners of these PH domains could be investigated in future studies.

The Phytozome database annotations show five of these proteins have no known functions; these are Cre02.g119150.t1.2, Cre03.g170650, Cre12.g525450.t1.1, Cre14.g614350.t1.2, and Cre14.g616050.t1.1. Among these, Cre12.g525450.t1.1 and Cre14.g616050.t1.1 are annotated as having mating activation-specific expression [33], suggesting these two proteins might have functions in the mating process. This is in correlation with the RNA-seq data of Strenkert et al. [34], which shows very little expression (having FPKM values close to zero in their studies) under autotrophic growth conditions for both Cre12.g525450.t1.1 and Cre14.g616050.t1.1. Additionally, according to Breker et al. [35], a null allele of Cre12.g525450 was isolated in their study and found to be non-essential in the cell cycle. These data all conclusively suggest that these two genes most likely have a function in the mating process. Interestingly, our BLAST search did not find any orthologs in *A. thaliana* and *V. carterii*, and the Phytozome database shows they both have orthologs in *Chlorella zofingiensis*, *Dunaliella salina*, and some other species. In future work, it will be interesting to investigate their specific functions and the role of the PH domain in the mating process by studying localization and protein–protein interaction. Cre02.g119150.t1.2, Cre03.g170650, and Cre14.g614350.t1.2 are shown to have transcript [35], suggesting these genes are active, although their function is unknown (Table 1).

The remaining proteins are annotated as having predicted functions according to their homology with other species’ proteins (Table 1). According to a search of the literature, there are no reported functional analyses of these proteins. Among these is the predicted Ca/calmodulin-dependent kinase (Cre03.g153150). According to Strenkert et al. [35], transcription of the predicted Ca/calmodulin-dependent kinase (Cre03.g153150) reaches high levels at the end of the light period, suggesting a role in the cell cycle process. Other genes have constitutive expression (not changing under light and dark conditions), suggesting these genes are active.

An RNA-seq study [36] that examined the transcriptional changes under phosphate deficiency shows that there is slight increase in transcription of Cre02.g119150 (1.88 fold in linear scale) (unknown function), Cre14.g616050 (1.26 fold in linear scale) (unknown function, mating-related), and Cre16.g653700 (1.82 fold in linear scale) (putative oxysterol binding protein, OSBP). Notably, the *psr1* mutant does not have an increase in transcription of these genes, suggesting that upregulation of these genes is PSR1-dependent, providing further evidence for regulation under phosphate deficiency. In contrast, there is decrease in Cre03.g170650 (0.7 fold in linear scale) (unknown function) and Cre12.g525450 (0.52 fold in linear scale) (unknown function, mating-related). In light of these results, it is plausible to suggest that the function of the putative OSBP could involve membrane remodeling and perhaps signaling under phosphate deficiency. It has been known for many years that there is a decrease in phospholipids and increase in non-phospholipids under phosphate deficiency [37]. OSPBs belong to a major and evolutionarily conserved family of lipid transfer proteins (LTPs). LTPs comprise OSBPs, OSPB-related proteins (ORPs) in higher eukaryotes, and oxysterol-binding homology (Osh) in yeast. A number of these were found to extract and replace sterol or phospatidylserine (PS) in exchange for PIPs [38]. Results of Bajhaiya et al. [36] also show that all the genes are transcribed and there is also regulation under phosphate deficiency.

In conclusion, our structure models and bioinformatic analysis along with expression data found in the literature provide possible roles of these gene models for PH-containing proteins in *C. reinhardtii*, an organism thought to retain the characteristics of the last eukaryotic common ancestor [39]. This opens new avenues for future studies to establish the function of PH domains in *C. reinhardtii*. Our results will be especially useful for mutation analysis of these genes in future studies, where we envision generating mutations in the PH domain-coding region of the genes using CRISPR or obtaining insertional mutants, which have insertions in or nearby PH domains, available from the CLIP library [40]. These mutants can then be screened for various phenotypes, such as mating capacity, and more precisely, if they have any defects in specific stages of the mating process. Other phenotypes such as defects in photosynthetic activity and acclimation to various stress conditions can also be screened. These phenotypic screens along with localization and protein–protein interaction studies in future studies could establish the foundation for complete functional annotation of the PH domain-containing proteins coded for by the *C. reinhardtii* genome.

## 4. Materials and Methods

### 4.1. Sequence Analysis

Initially, the full-length amino acid sequences of PH domain-containing proteins from *Chlamydomonas reinhardtii* were identified using similarity searches, keyword searches against the UniProt [41] and NCBI [42] protein sequence databases, and domain-based searches of the SMART [43] domain database, restricting the search to *Chlamydomonas reinhardtii*. Using amino acid sequences of 10 identified PH domain-containing proteins, Cre02.g079550, Cre02.g119150, Cre03.g153150,Cre03.g154150, Cre03.g170650, Cre04.g229163, Cre12.g548900, Cre14.g614350, Cre14.g616050, and Cre16.g653700, further information was retrieved from the publicly available databases Phytozome [25] and Algae Path [26]. The sequence of the eleventh PH domain-containing protein, Cre12.g525450, was identified using Hidden Markov Models (HMMER) using its default parameters [44]. The sequences of the PH domains for the identified proteins are provided in Supplementary Table S3. Cellular localization was predicted using two algorithms, PredAlgo [45] and DeepLoc [46]. Molecular weight and isoelectric point were computed using the Expasy database program, Compute pI/MW [47].

### 4.2. Domain Architecture Analysis

The domain architecture of the PH domain-containing proteins was analyzed by the SMART [43], Prosite [48], PFAM [49], InterPro [50], and CDD [51] databases. The consensus of the analysis for each of the individual proteins was mapped to a domain architecture cartoon using the IBS webserver [52].

### 4.3. Secondary Structure Prediction (SSP)

The predicted secondary structure of the PH domain was generated using PsiPred [53], PSSpred [54], SABLE [55], and PHD server [56]. The consensus of the predictions was derived and visualized using GeneDoc [57]. The domain boundaries were further refined using the SSP results and PH domains extracted from the sequences based on this refinement. The predicted structures were analyzed to assess their consistency with the canonical PH domain fold, characterized by a β-sandwich composed of seven β-strands and a C-terminal α-helix.

### 4.4. Three-Dimensional Modeling

Tertiary structure models of the extracted PH domain sequences were generated using homology- and ab initio-based modeling platforms, including SwissModel [58], Phyre2 [59], IntFold [60], I-Tasser [61], Robetta [62], Modeller [63], and QUARK [64]. Additionally, the 3D models were also developed using the AlphaFold3 webserver [65], as it became available after the study had been initiated. To identify the highest-quality tertiary structure model for each PH domain, the structural quality of the top-scoring models was evaluated using model validation tools such as ProSa-web [66], VoroMQA [67], Verify3D [68], QMEAN [69], and ModFold [70].

### 4.5. Biophysical Characterization of the Models

The top-ranking 3D models were characterized by mapping their surface electrostatic potentials in PyMol [71] using the APBS plugin [72] with the molecular surface visualization range set at (−4 to +4 kT/e), and visualized as a mesh over the C-alpha trace of the PH domain.

### 4.6. Docking

Each PH domain 3D model was subjected to molecular docking against a panel of seven phosphoinositide ligands: Phosphatidylinositol 3-phosphate (PI(3)P), Phosphatidylinositol 4-phosphate (PI(4)P), Phosphatidylinositol 5-phosphate, PI(5)P, Phosphatidylinositol 3,4-bisphosphate (PI(3,4)P2), Phosphatidylinositol 3,5-bisphosphate (PI(3,5)P2), Phosphatidylinositol 4,5-bisphosphate, (PI(4,5)P2), and Phosphatidylinositol 3,4,5-trisphosphate (PI(3,4,5)P3). Protein–ligand docking utilized HDock [73], a hybrid algorithm of template-based modeling and ab initio free docking. To identify interface residues and characterize the types of molecular interactions involved in ligand binding, the top-ranked docking scenarios were selected, and the PDB files were saved and analyzed in Liglot+ [74] via PDBSum [75].

### 4.7. Multiple Sequence Alignment (MSA)

Since PH domains typically share very low sequence similarity, a structure-based multiple sequence alignment of the PH domains was performed by superposition of the PH domains in SuperPose [76]. The MSA was visualized using ESPript 3 [77] and annotated to map the lipid-binding residues identified in the docking predictions.

### 4.8. Phylogeny Analysis

Putative orthologs in *A. thaliana* and *V. carterii* were screened in the sequence databases using a BLAST search. Domain analysis was then performed for each ortholog. Only the orthologs containing PH domains were included in the phylogeny analysis. A phylogenetic tree was generated using ClustalW (https://www.genome.jp/tools-bin/clustalw) (accessed on 6 August 2025). Alignments and phylogenetic reconstructions were performed using the function “build” in ETE3 v3.1.1 [78], as implemented in GenomeNet (https://www.genome.jp/tools/ete/) (accessed on 6 August 2025). The phylogenetic tree was constructed using fasttree with slow NNI and MLACC = 3 (to make the maximum-likelihood NNIs more exhaustive) [79]. Values at nodes are SH-like local supports.

## Figures and Tables

**Table 1:** Protein sequence analysis and cellular localization predictions. pI—isoelectric point, MW—molecular weight, other—cellular localization other than chloroplast, mitochondria, or secretory pathway, NLS—nuclear localization signal.

Gene Model	Annotation (Phytozome)	pI/MW (Residues)	Predicted Localization PredAlgo (DeepLoc)
Cre02.g079550.t1.2	Dynamin GTPase	8.87/91,230.45 (868)	Other (Cytoplasm, NLS)
Cre02.g119150.t1.2	No known function (Hypothetical, found in green algae only)	5.56/73,642.51 (698)	Other (Cytoplasm-NLS)
Cre03.g153150	Calcium/calmodulin-dependent protein kinase	10.40/257,848.56 (2667)	Mitochondria (Cytoplasm, NLS)
Cre03.g154150.t1.1	Arf GAP	6.68/94,698.00 (907)	Other (Cytoplasm)
Cre03.g170650	No known function (found in green algae and charophyte)	6.25/67,312.52 (636)	Other (Cytoplasm, NLS)
Cre04.g229163	Formin like, involved in cytoskeletal rearrangement.	6.67/259,728.68 (2683)	Other (Cytoplasm)
Cre12.g525450.t1.1	No known function (mating activation-specific expression in plus gametes; found in green algae only)	9.48/239,756.79 (2372)	Other (Cytoplasm, NLS)
Cre12.g548900.t1.1	ROC23, circadian rhythm-related (Rho GTPase activating protein)	7.73/55,982.70 (531)	Other (Cytoplasm, Lysosome/Vacuole, NLS)
Cre14.g614350.t1.2	No known function	8.45/16,209.40 (141)	Other (Cytoplasm-NLS)
Cre14.g616050.t1.1	No known function (mating activation-specific expression)	9.83/117,412.88 (1160)	Mitochondria (Cytoplasm)
Cre16.g653700	Oxysterol binding	6.02/127,272.95 (1218)	Mitochondria (Cytoplasm)

**Table 2:** Phosphoinositide binding profile of the Chlamydomonas PH domains. The highest-scoring phosphoinositide interaction for each PH domain is marked with an asterisk.

PH Domain	Phosphoinositide	Residues Involved in Binding
Cre02.g079550.t1.2	I4P *	Gln 57, Asn 59, His 98, Val 104
Cre02.g119150.t1.2	I3,4P *	Ala 58, Glu 79, Arg 80
	I3P	Lys 9, Arg 80, Val 81
	I5P	Ala 58, Glu 79
	I4,5P	Lys 9, Arg 80, Arg 83
	I3,4,5P	Lys 9, Lys 12, Gln 24, Arg 26
Cre03.g153150	I3P *	Lys 10, Ser 12, Lys 81
Cre03.g154150.t1.1	I3,4,5P *	Lys 10, Arg 33, Thr 67
	I3P	Lys 10, Arg 33, Thr 66, Thr 67
	I4P	Lys 10, Arg 33, Thr 66
	I5P	Lys 10, Arg 33, Thr 66
	I3,4P	Lys 10, Arg 33, Thr 66, Thr 67
Cre03.g170650	IPA *	Gly 45
	I3P	Arg 19, Arg 79
	I4P	Arg 79
	I5P	Trp 33, Arg 79
	I3,4P	Trp 11, Arg 79, Ser 82
	I4,5P	Arg 19, Cys 21, Trp 33, Arg 79
	I3,4,5P	Arg 19, Cys 21, Trp 33
Cre04.g229163	IPA *	Lys 11, Arg 19
	I3P	Lys 11, His 79
	I4P	Trp 78
	I5P	Thr 76, Trp 78
	I3,4P	Lys 73, Trp 78
	I4,5P	Lys 11, Arg 77, His 79
	I3,4,5P	Val 48, Thr 76, Trp 78
Cre12.g525450	IPA *	Arg 18, Ser 40, Tyr 67
	I3P	Arg 18, Ser 40, Tyr 67
	I5P	Arg 18, Ser 40
	I3,4P	Lys 9, Arg 18, Ser 40, Tyr 67
	I4,5P	Lys 9, Arg 18, Ser 40, Tyr 67
	I3,4,5P	Lys 9, Arg 18, Arg 39, Ser 40, Tyr 67
Cre12.g548900	IPA *	Gln 11, His 77
	I3P	Arg 32, His 77
	I4P	Ser 10, Gln 11, Arg 32, Hys 77, Tyr 78
	I5P	Ser 10
	I3,4P	Gln 11, Lys 16, Arg 32, Val 47, His 77
	I4,5P	Ser 10, Asp 13, Arg 21, Arg 32
	I3,4,5P	Ser 10, Gln 11, Val 47, Tyr 78
Cre14.g614350	I3,4,5P *	Lys 10, Arg 19, Arg 21
	I3P	Lys 10, Arg 19, Arg 21
	I4P	Lys 10, Glu 13, Trp 32
	I5P	Lys 10, Arg 19, Arg 21
	I3,4P	Arg 19, Arg 21, Arg 45
	IPA	Lys 10
Cre14.g616050	IPA *	Lys 10, Arg21, Arg 43
	I3P	Ser 83, Arg 84
	I4P	Lys 10, Ser 83, Arg 84
	I5P	Trp 32, Ser 83, Arg 84
	I3,4P	Lys10, Arg 43, Ser 83, Arg 84
	I4,5P	Lys 10
	I3,4,5P	Lys 10, Arg 21, Arg 43
Cre16.g653700	IPA *	His 24
	I5P	Arg 26, Lys 41, Trp 50
	I3,4P	Arg 26, Lys 41, Lys 48
	I3,4,5P	Arg 26

## Data Availability

The data presented in this study are available in the article and the Chlamydomonas sequences are publicly available.

## Supplementary Materials

The following supporting information can be downloaded at: https://www.mdpi.com/article/10.3390/plants14172607/s1, Table S1: Summary of the evaluation scores of PH domain 3D models generated by different modeling servers evaluated by ProSa-web, VoroMQA, Verify3D, QMEAN, and ModFold. The model quality is depicted on a color scale from dark blue indicating the highest quality to yellow indicating the lowest quality; Table S2: Comparison of interaction scenarios of wild type versus mutated *C. reinhardtii* PH domains. HB stands for number of hydrogen bonds stabilizing the complex. The sequences displaying binding in non-canonical pockets are marked with an asterisk next to the sequence name; Table S3: Amino acid sequences of the identified PH domains from the *C. reinhardtii* proteins; Figure S1. Surface electrostatics and binding profiles of the top PH domain models. A. Surface electrostatic potentials of PH domain color-graded from −4 kT/e (red) to +4 kT/e (blue). B. Mesh presentation electrostatic potential, color-graded from −4 kT/e (red) to +4 kT/e (blue). C–F. Binding scenarios with phosphoinositides.
