# Supplementary material for: A Genome-Wide Modeling and Characterization Study of Pleckstrin Homology Domains in Chlamydomonas reinhardtii
Source: Plants (Basel). 2025 Aug 22;14(17):2607. doi: 10.3390/plants14172607 (PMC12430284; doi:10.3390/plants14172607)
Supplement: Supplementary file 1 [file plants-14-02607-s001.zip › plants-3749401-supplementary.pdf]

Table S1. Summary of the evaluation scores of PH domain 3D models generated by different modeling servers evaluated by ProSa-web, VoroMQA, Verify3D, QMEAN, and ModFold. The model quality is depicted on a color scale from dark blue indicating the highest quality to yellow indicating the lowest quality.

	Swiss Model	Phyre2	IntFold	ITasser	Robetta	Modeller	QUARK	Alpha Fold
**Cre02.g079550.t1.2**	✖ blue	✖ green	✖ yellow	✖ light green	✖ dark blue	✖ yellow	✖ yellow	✖ dark blue
**Cre02.g119150.t1.2**	✖ blue	✖ yellow	✖ yellow	✖ yellow	✖ dark blue	✖ blue	✖ green	✖ blue
**Cre03.g153150**	✖ green	✖ light green	✖ dark blue	✖ blue	✖ dark blue	✖ yellow	✖ green	✖ blue
**Cre03.g154150.t1.1**	✖ green	✖ light green	✖ blue	✖ blue	✖ dark blue	✖ green	✖ yellow	✖ green
**Cre03.g170650**	✖ dark blue	✖ blue	✖ blue	✖ green	✖ dark blue	✖ green	✖ green	✖ blue
**Cre04.g229163**	✖ dark blue	✖ dark green	✖ dark green	✖ blue	✖ blue	✖ green	✖ dark green	✖ dark green
**Cre12.g525450**	✖ dark green	✖ dark green	✖ blue	✖ blue	✖ dark blue	✖ green	✖ yellow	✖ blue
**Cre12.g548900.t1.1**	✖ blue	✖ dark blue	✖ blue	✖ dark green	✖ dark blue	✖ green	✖ dark green	✖ blue
**Cre14.g616050.t1.1**	✖ blue	✖ dark green	✖ blue	✖ blue	✖ dark blue	✖ dark green	✖ blue	✖ blue
**Cre14.g614350.t1.2**	✖ blue	✖ dark green	✖ blue	✖ blue	✖ dark blue	✖ dark green	✖ blue	✖ blue
**Cre16.g653700**	✖ dark blue	✖ dark green	✖ blue	✖ blue	✖ dark blue	✖ blue	✖ green	✖ dark blue

Table S2. Comparison of interaction scenarios of wild type versus mutated *C. reinhardtii* PH domains. HB stands for number of hydrogen bonds stabilizing the complex. The sequences displaying binding in non – canonical pockets are marked with an asterisk next to the sequence name

	HB	Key interacting Residues	Mutations	HB	Key interacting residues
**Cre03.g153150**	4	Lys 10, Ser 12, Lys 81	Ala 10, Ala 12, Ala 81	1	Tyr 32
**Cre03.g154150.t1.1**	6	Lys 10, Arg 33, Thr 66	Ala 10, Ala 33, Ala 66	3	Ala 10, Arg 11, Lys 31
**Cre04.g229163**	3	Lys 11, Arg 19	Ala 11, Ala 19	n/a	n/a
**Cre14.g614350.t1.2**	5	Lys 10, Arg 19, Arg 21	Ala 10, Ala 19, Ala 21	2	Glu 13, Trp 32
**Cre14.g616050.t1.1**	5	Lys 10, Arg 21, Arg 43	Ala 10, Ala 21, Ala 43	0	n/a
**Cre16.g653700**	3	His 24	Ala 24	n/a	n/a
**Cre02.g079550.t1.2***	4	Gln 57, Asn 59, His 98, Val 104	Ala 57, Ala 59, Ala 98, Ala 10	n/a	n/a
**Cre02.g119150.t1.2***	3	Ala 58, Glu 79, Arg 80	Ala 58, Ala 79, Ala 80	5	Lys 9, Arg 26, Glu 55
**Cre03.g170650***	1	Gly 45	Ala 45	1	Ala 45
**Cre12.g525450***	4	Arg 18, Ser 40, Tyr 67	Ala 18, Ala 40, Ala 67	2	Lys 9, Arg 39
**Cre12.g548900.t1.1 ***	2	Gln 11, His 77	Ala 11, Ala 77	4	Ser 10, Ala 11, Arg 32, Tyr 78

Table S3. Amino acid sequences of the identified PH domains from the *C. reinhardtii* proteins.

	PH Domain Sequence
Cre02.g079550.t1.2	DDFIAGYFDKYVSDDSARFLESMKWQRRFFVFSESQRVLYYFKSPEDVSKPSGLRGQVNIAECLVEDLDDKGNARPVGAGPATLNPLDKGQLMIRIRHKDPRGVAVKDHNAIIMRAENIDTKMTWLGKLRKAADPR
Cre02.g119150.t1.2	IIIDGFLLKKKKDDKRSFLTSKYQRRYFELTSDTLLYAKDPKDLKDLKDGSGDIEVFAIHELKYIKKLEDEKLEMKFPERVLRVKAESKADHERWYEAIRDARQKK
Cre03.g153150	SPVRHGFLWKRSRAFKSWLSRWFQLDDTGFTYAVSPKLIKREGRYVLPSAVREVLPLPPERYRNGATMYGVRVVATSGATKDLYTDDAAALEAWTRDLARSTPAA
Cre03.g154150.t1.1	TVLRQGYLLKRSGGAGAGGGAGGKVVAGEWKRRFFVLDSRGVLYYYSQKDSLLNKLRGVEAHTPATTGVNLLTSTIKLDDEAEPQLRFCFRVVSPTGTLALQAESEPDRAAWVSLLQVAISTL
Cre03.g170650	EISRQGDLQVWEPATERWRKCHFVLTRAGYLHWFPKAEEVRPLDGLALARCAFEAGKAPRFNIIETAKGGGWLGGIRGRRLSFQAASVEECCEWAIAIREAIAVA
Cre04.g229163	ALAVKSGLLWKQDTWSFWRKHFYVIFAGEEPAIALYYQEKIDARPDKVMPLPGCRVLDILEQQGRFKFTLEFKDKTRWHLASQSGDDRKEWISTIVPLAAGA
Cre12.g525450	LLLKGPVQKKTMRGFQDRYLILVPRKLYIMSSKTAIYPRSVLSLLDANPRYDEARGTVHLDVMGKEYVFRATGGGTAAGGGGSGGGGGGGGEGHSREETLEWFVALAHGCGLP
Cre12.g548900.t1.1	SPDKAGWLQSQGDVIKNWRNRWFVLKQGYLFRFYNDKVAESIKPRGVVDLSKVQDVKVLPGRGNTIQLKTTSGGVVHYIASTETEVVEWVSAIEGAMAKI
Cre14.g614350.t1.2	SPERCGWLQKQGEIIKTWRRRWFVMKQGKIFWFKSDVVTPDSVPRGVIDVNKCLSIKGAEDTINKANAFEISTQTESMFFIADTDKEKEDWINAIGRAIVKH
Cre14.g616050.t1.1	PPDLSGWLDKEGRRSTAWKRRFFILKGSNFFWFEGQGGGVRPRGFIALEGSSVACRTVVNRTGEKPFALTITLPPEAAEGGASRPHLTVAAVSEELQSLWFRALSQAAIPR
Cre16.g653700	RLKRDGMPPASYMWLWLGNFKRWHKRYFVASEAPGVLLIYKRANMKGKVWSTSLVDATVAQDDSHPRQIRLATPSGTIFLRVLRPEERQPWLACLRDSVATY

Cre02.g079550.t1.2

A

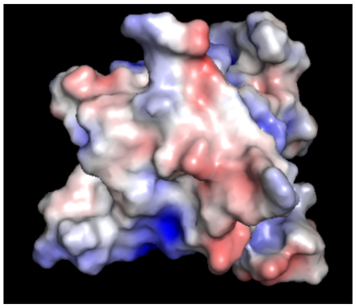

B

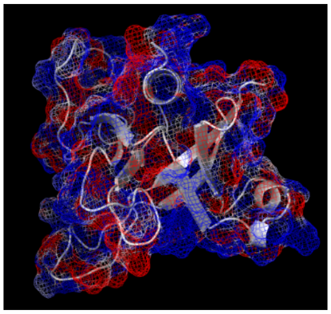

C

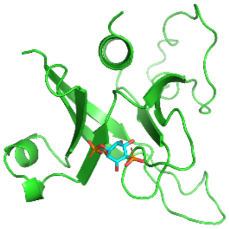

PDB code: qew7   Ligand PDB

Hydrogen bonds

	<----- A T O M 1 ----->						<----- A T O M 2 ----->					
	Atom no.	Atom name	Res name	Res no.	Chain		Atom no.	Atom name	Res name	Res no.	Chain	Distance
1.	932	NE2	GLN	57	A	-->	21	O11	PDB	1		2.59
2.	957	N	ASN	59	A	-->	25	O2	PDB	1		3.04
3.	1555	NE2	HIS	98	A	-->	29	O3	PDB	1		2.52
4.	1642	N	VAL	104	A	-->	5	O4	PDB	1		3.20

Cre02.g119150.t1.2

A

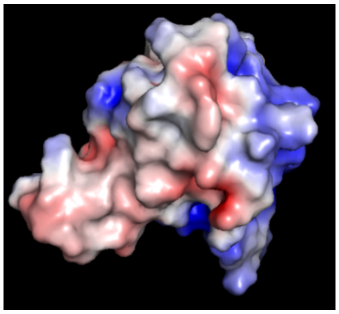

B

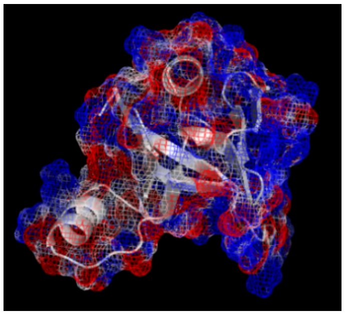

C

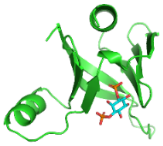

Hydrogen bonds

	<----- A T O M 1 ----->						<----- A T O M 2 ----->					
	Atom no.	Atom name	Res name	Res no.	Chain		Atom no.	Atom name	Res name	Res no.	Chain	Distance
1.	145	NZ	LYS	9	A	-->	5	O1	PDB	1		2.66
2.	1366	NE	ARG	80	A	-->	29	O6	PDB	1		3.26
3.	1383	N	VAL	81	A	-->	21	O4	PDB	1		3.12

D

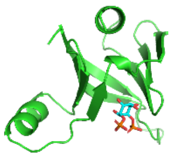

Hydrogen bonds

	<----- ATOM 1 -----> Atom no.	Atom name	Res name	Res no.	Chain		<----- ATOM 2 -----> Atom no.	Atom name	Res name	Res no.	Chain	Distance
1.	968	N	ALA	58	A	-->	16	O12	PDB	1		2.35
2.	1344	N	GLU	79	A	-->	21	O4	PDB	1		3.20

E

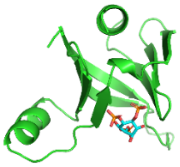

Hydrogen bonds

	<----- ATOM 1 -----> Atom no.	Atom name	Res name	Res no.	Chain		<----- ATOM 2 -----> Atom no.	Atom name	Res name	Res no.	Chain	Distance
1.	968	N	ALA	58	A	-->	4	O5P	PDB	1		3.10
2.	1344	N	GLU	79	A	-->	32	O7P	PDB	1		3.31
3.	1366	NE	ARG	80	A	-->	16	O2P	PDB	1		2.99

F

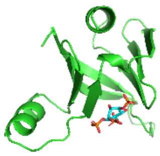

Hydrogen bonds

	<----- ATOM 1 -----> Atom no.	Atom name	Res name	Res no.	Chain		<----- ATOM 2 -----> Atom no.	Atom name	Res name	Res no.	Chain	Distance
1.	145	NZ	LYS	9	A	-->	25	O6	PDB	1		2.56
2.	1366	NE	ARG	80	A	-->	14	O2	PDB	1		2.65
3.	1418	N	ARG	83	A	-->	33	O53	PDB	1		3.04

G

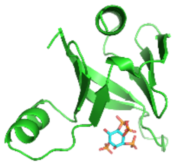

PDB code: ror2   Ligand PDB

Hydrogen bonds

	<----- ATOM 1 -----> Atom no.	Atom name	Res name	Res no.	Chain		<----- ATOM 2 -----> Atom no.	Atom name	Res name	Res no.	Chain	Distance
1.	145	NZ	LYS	9	A	-->	3	OP5	PDB	1		2.28
2.	203	N	LYS	12	A	-->	36	OP8	PDB	1		2.59
3.	421	NE2	GLN	24	A	-->	23	OP1	PDB	1		3.31
4.	461	NE	ARG	26	A	-->	17	O2	PDB	1		2.66
5.	464	NH2	ARG	26	A	-->	17	O2	PDB	1		2.92
6.	464	NH2	ARG	26	A	-->	23	OP1	PDB	1		2.68

H

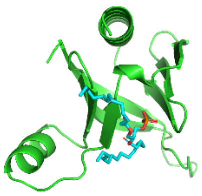

Cre03.g153150

A

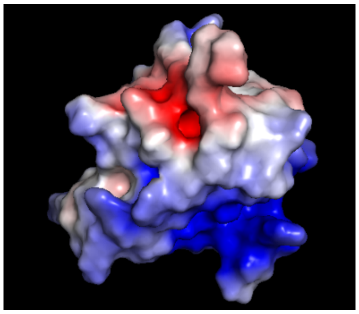

B

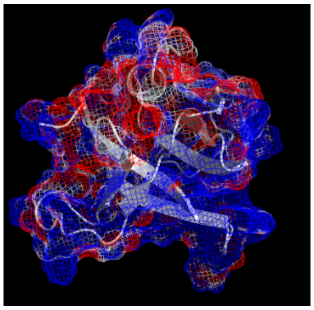

C

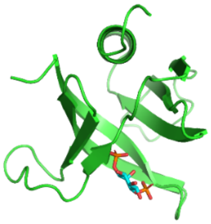

PDB code: smh7   Ligand PDB

Hydrogen bonds

	ATOM 1 Atom no.	ATOM 1 Atom name	ATOM 1 Res name	ATOM 1 Res no.	ATOM 1 Chain		ATOM 2 Atom no.	ATOM 2 Atom name	ATOM 2 Res name	ATOM 2 Res no.	ATOM 2 Chain	Distance
1.	163	NZ	LYS	10	A	-->	4	OP2	PDB	1		2.44
2.	206	OG	SER	12	A	-->	3	OP3	PDB	1		3.05
3.	1322	NZ	LYS	81	A	-->	1	OP1	PDB	1		3.04
4.	1322	NZ	LYS	81	A	-->	10	O2	PDB	1		2.62

Cre03.g154150

A

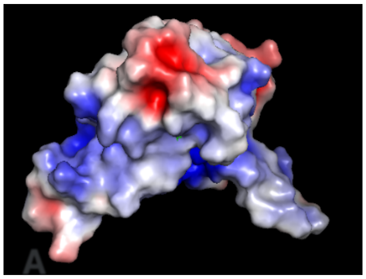

B

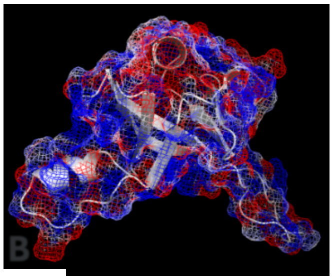

C

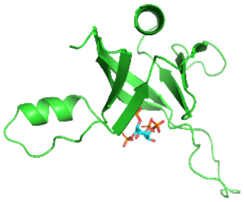

Hydrogen bonds

	ATOM 1 Atom no.	ATOM 1 Atom name	ATOM 1 Res name	ATOM 1 Res no.	ATOM 1 Chain		ATOM 2 Atom no.	ATOM 2 Atom name	ATOM 2 Res name	ATOM 2 Res no.	ATOM 2 Chain	Distance
1.	167	NZ	LYS	10	A	-->	23	OP1	PDB	1		2.18
2.	465	NE	ARG	33	A	-->	1	OP4	PDB	1		2.69
3.	465	NE	ARG	33	A	-->	36	OP8	PDB	1		2.78
4.	468	NH2	ARG	33	A	-->	3	OP5	PDB	1		2.22
5.	468	NH2	ARG	33	A	-->	13	O12	PDB	1		2.55
6.	1021	OG1	THR	67	A	-->	25	OP3	PDB	1		2.70

D

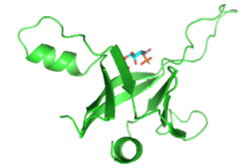

PDB code: rbu9 Ligand PDB

Hydrogen bonds

	<----- A T O M 1 ----->						<----- A T O M 2 ----->					
	Atom no.	Atom name	Res name	Res no.	Chain		Atom no.	Atom name	Res name	Res no.	Chain	Distance
1.	167	NZ	LYS	10	A	-->	1	OP1	PDB	1		3.01
2.	465	NE	ARG	33	A	-->	14	O3	PDB	1		3.20
3.	1002	N	THR	66	A	-->	18	O10	PDB	1		3.27
4.	1007	OG1	THR	66	A	-->	18	O10	PDB	1		1.90
5.	1007	OG1	THR	66	A	-->	21	O4	PDB	1		2.93
6.	1016	N	THR	67	A	-->	18	O10	PDB	1		2.67

E

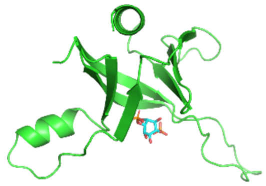

PDB code: rbw1 Ligand PDB

Hydrogen bonds

	<----- A T O M 1 ----->						<----- A T O M 2 ----->					
	Atom no.	Atom name	Res name	Res no.	Chain		Atom no.	Atom name	Res name	Res no.	Chain	Distance
1.	167	NZ	LYS	10	A	-->	10	O5	PDB	1		2.80
2.	465	NE	ARG	33	A	-->	18	O1	PDB	1		2.90
3.	1007	OG1	THR	66	A	-->	21	O11	PDB	1		2.72

F

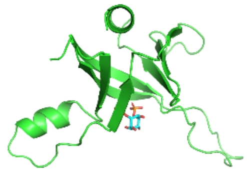

PDB code: qto5 Ligand PDB

Hydrogen bonds

	<----- A T O M 1 ----->						<----- A T O M 2 ----->					
	Atom no.	Atom name	Res name	Res no.	Chain		Atom no.	Atom name	Res name	Res no.	Chain	Distance
1.	167	NZ	LYS	10	A	-->	10	O6	PDB	1		2.75
2.	465	NE	ARG	33	A	-->	5	O1	PDB	1		2.67
3.	465	NE	ARG	33	A	-->	14	O5	PDB	1		2.94
4.	465	NE	ARG	33	A	-->	25	O3	PDB	1		2.81
5.	468	NH2	ARG	33	A	-->	5	O1	PDB	1		2.77
6.	468	NH2	ARG	33	A	-->	25	O3	PDB	1		2.37
7.	1007	OG1	THR	66	A	-->	16	O53	PDB	1		2.18

G

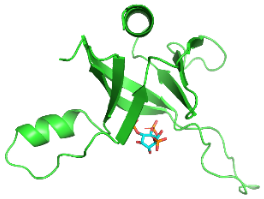

PDB code: qto9 Ligand PDB

Hydrogen bonds

	<----- A T O M 1 ----->						<----- A T O M 2 ----->					
	Atom no.	Atom name	Res name	Res no.	Chain		Atom no.	Atom name	Res name	Res no.	Chain	Distance
1.	167	NZ	LYS	10	A	-->	5	O3	PDB	1		2.95
2.	167	NZ	LYS	10	A	-->	10	O2	PDB	1		2.42
3.	465	NE	ARG	33	A	-->	1	O4P	PDB	1		2.75
4.	468	NH2	ARG	33	A	-->	1	O4P	PDB	1		2.88
5.	1007	OG1	THR	66	A	-->	25	O5	PDB	1		2.59
6.	1021	OG1	THR	67	A	-->	17	O3P	PDB	1		2.51

Cre03.g170650

A

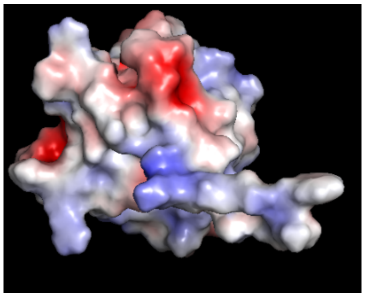

B

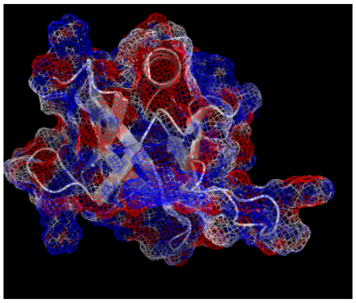

C

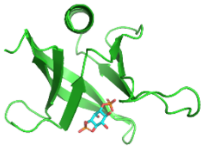

Hydrogen bonds

	Atom no. (Atom 1)	Atom name	Res name	Res no.	Chain		Atom no. (Atom 2)	Atom name	Res name	Res no.	Chain	Distance
1.	165	NH1	ARG	19	A	-->	3	OP3	PDB	1		2.55
2.	633	NH2	ARG	79	A	-->	21	O4	PDB	1		2.81

D

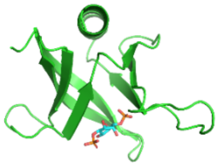

PDB code: sxn9 Ligand PDB

Hydrogen bonds

	Atom no. (Atom 1)	Atom name	Res name	Res no.	Chain		Atom no. (Atom 2)	Atom name	Res name	Res no.	Chain	Distance
1.	633	NH2	ARG	79	A	-->	10	O5	PDB	1		2.41

E

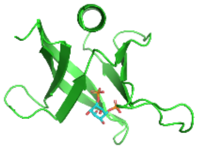

PDB code: syl9 Ligand PDB

Hydrogen bonds

	Atom no. (Atom 1)	Atom name	Res name	Res no.	Chain		Atom no. (Atom 2)	Atom name	Res name	Res no.	Chain	Distance
1.	283	NE1	TRP	33	A	-->	21	O4	PDB	1		2.98
2.	633	NH2	ARG	79	A	-->	10	O6	PDB	1		2.32

F

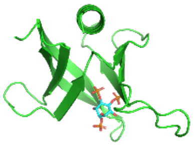

Hydrogen bonds

	<----- A T O M 1 ----->						<----- A T O M 2 ----->					
	Atom no.	Atom name	Res name	Res no.	Chain		Atom no.	Atom name	Res name	Res no.	Chain	Distance
1.	80	N	TRP	11	A	-->	33	O9P	PDB	1		3.28
2.	633	NH2	ARG	79	A	-->	21	O6	PDB	1		2.77
3.	653	N	SER	82	A	-->	1	O4P	PDB	1		2.72
4.	653	N	SER	82	A	-->	33	O9P	PDB	1		2.66

G

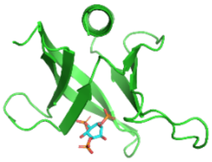

PDB code: rih9 Ligand PDB

Hydrogen bonds

	<----- A T O M 1 ----->						<----- A T O M 2 ----->					
	Atom no.	Atom name	Res name	Res no.	Chain		Atom no.	Atom name	Res name	Res no.	Chain	Distance
1.	165	NH1	ARG	19	A	-->	32	O52	PDB	1		2.67
2.	181	SG	CYS	21	A	-->	31	O51	PDB	1		2.68
3.	283	NE1	TRP	33	A	-->	29	O5	PDB	1		2.57
4.	633	NH2	ARG	79	A	-->	10	O3	PDB	1		1.96

H

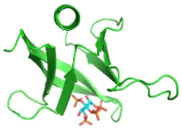

PDB code: rii1 Ligand PDB

Hydrogen bonds

	<----- A T O M 1 ----->						<----- A T O M 2 ----->					
	Atom no.	Atom name	Res name	Res no.	Chain		Atom no.	Atom name	Res name	Res no.	Chain	Distance
1.	165	NH1	ARG	19	A	-->	3	OP5	PDB	1		2.70
2.	165	NH1	ARG	19	A	-->	13	O12	PDB	1		2.91
3.	181	SG	CYS	21	A	-->	4	OP6	PDB	1		2.93
4.	283	NE1	TRP	33	A	-->	5	O4	PDB	1		3.13

I

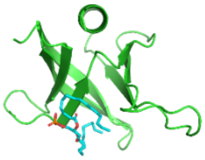

PDB code: pbi3 Ligand PDB

Hydrogen bonds

	<----- A T O M 1 ----->						<----- A T O M 2 ----->					
	Atom no.	Atom name	Res name	Res no.	Chain		Atom no.	Atom name	Res name	Res no.	Chain	Distance
1.	380	N	GLY	45	A	-->	45	O22	PDB	1		2.77

Cre04.g229163

A

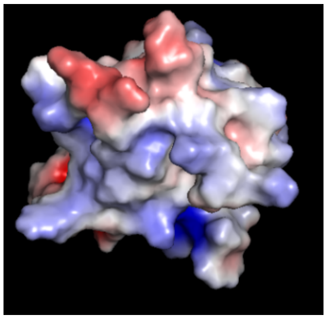

B

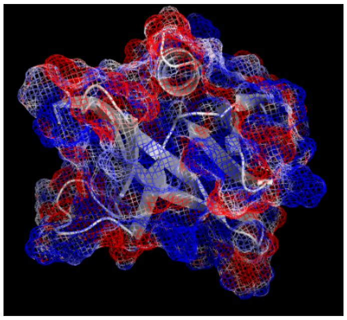

C

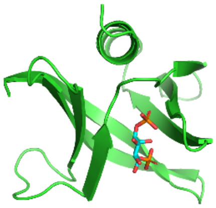

```
PDB code: qxo9    Ligand PDB
--------------------------

Hydrogen bonds
--------------

       <----- A T O M   1 ----->       <----- A T O M   2 ----->

       Atom Atom Res   Res             Atom Atom Res   Res
        no. name name no.  Chain        no. name name no.  Chain  Distance
  1.    168  NZ  LYS   11    A    -->     4  OP2 PDB    1            2.28
  2.   1326  N   HIS   79    A    -->    16  O12 PDB    1            3.21
```

D

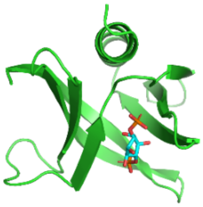

```
PDB code: qxp4    Ligand PDB
--------------------------

Hydrogen bonds
--------------

       <----- A T O M   1 ----->      <----- A T O M   2 ----->

       Atom Atom Res   Res            Atom Atom Res   Res
        no. name name no.  Chain       no. name name no.  Chain  Distance
  1.   1310  NE1 TRP   78    A    -->    3  O43 PDB    1            2.56
```

E

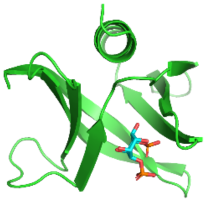

```
PDB code: qzq8    Ligand PDB
--------------------------

Hydrogen bonds
--------------

       <----- A T O M   1 ----->     <----- A T O M   2 ----->

       Atom Atom Res   Res           Atom Atom Res   Res
        no. name name no.  Chain      no. name name no.  Chain  Distance
  1.   1264  N   THR   76    A   -->    4  O12 PDB    1            3.14
  2.   1269  OG1 THR   76    A   -->    1  O11 PDB    1            2.86
  3.   1310  NE1 TRP   78    A   -->    3  O13 PDB    1            3.16
```

F

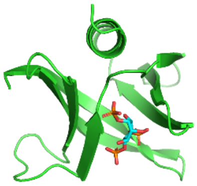

```
PDB code: rbu5    Ligand PDB
--------------------------

Hydrogen bonds
--------------

       <----- A T O M   1 ----->       <----- A T O M   2 ----->

       Atom Atom Res   Res             Atom Atom Res   Res
        no. name name no.  Chain        no. name name no.  Chain  Distance
  1.   1216  NZ  LYS   73    A    -->    16  O2P PDB    1            2.64
  2.   1310  NE1 TRP   78    A    -->     4  O5P PDB    1            2.68
```

G

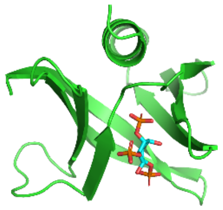

```
PDB code: syo3   Ligand PDB
---------------------------

Hydrogen bonds
--------------

      <----- A T O M   1 ----->       <----- A T O M   2 ----->

      Atom Atom Res  Res               Atom Atom Res  Res
       no. name name no.  Chain         no. name name no.  Chain  Distance
  1.   168  NZ  LYS   11    A    -->    22  O13 PDB    1            2.50
  2.  1278  N   ARG   77    A    -->     4  O42 PDB    1            3.25
  3.  1326  N   HIS   79    A    -->    31  O51 PDB    1            2.73
```

H

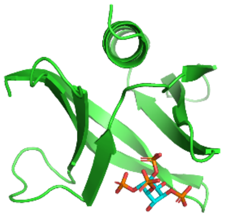

```
PDB code: rcp1   Ligand PDB
---------------------------

Hydrogen bonds
--------------

      <----- A T O M   1 ----->       <----- A T O M   2 ----->

      Atom Atom Res  Res               Atom Atom Res  Res
       no. name name no.  Chain         no. name name no.  Chain  Distance
  1.   791  N   VAL   48    A    -->     4  OP6 PDB    1            3.30
  2.  1269  OG1 THR   76    A    -->    10  O3  PDB    1            3.16
  3.  1269  OG1 THR   76    A    -->    17  O2  PDB    1            2.97
  4.  1269  OG1 THR   76    A    -->    21  O1  PDB    1            2.42
  5.  1269  OG1 THR   76    A    -->    23  OP1 PDB    1            2.40
  6.  1310  NE1 TRP   78    A    -->    14  O11 PDB    1            2.58
```

I

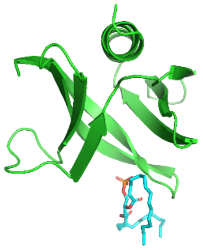

```
PDB code: pbj6   Ligand PDB
---------------------------

Hydrogen bonds
--------------

      <----- A T O M   1 ----->       <----- A T O M   2 ----->

      Atom Atom Res  Res               Atom Atom Res  Res
       no. name name no.  Chain         no. name name no.  Chain  Distance
  1.   168  NZ  LYS   11    A    -->    55  O14 PDB    1            2.14
  2.   313  NH1 ARG   19    A    -->    56  O13 PDB    1            1.88
  3.   314  NH2 ARG   19    A    -->    62  O32 PDB    1            2.79
```

Cre12.g525450

A

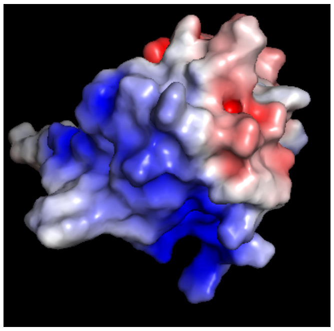

B

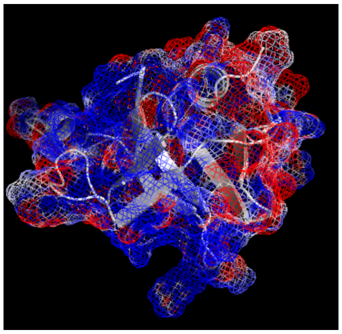

C

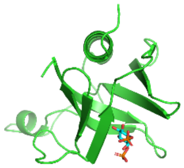

PDB code: rhb9 Ligand PDB

Hydrogen bonds

	ATOM 1						ATOM 2					
	Atom no.	Atom name	Res name	Res no.	Chain		Atom no.	Atom name	Res name	Res no.	Chain	Distance
1.	300	NH1	ARG	18	A	-->	29	O6	PDB	1		2.06
2.	301	NH2	ARG	18	A	-->	3	OP3	PDB	1		2.66
3.	696	OG	SER	40	A	-->	10	O2	PDB	1		2.36
4.	696	OG	SER	40	A	-->	17	O11	PDB	1		1.84
5.	1115	OH	TYR	67	A	-->	14	O3	PDB	1		2.79

D

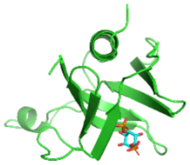

PDB code: rnk6 Ligand PDB

Hydrogen bonds

	ATOM 1						ATOM 2					
	Atom no.	Atom name	Res name	Res no.	Chain		Atom no.	Atom name	Res name	Res no.	Chain	Distance
1.	144	NZ	LYS	9	A	-->	10	O6	PDB	1		2.66
2.	696	OG	SER	40	A	-->	14	O5	PDB	1		2.53
3.	696	OG	SER	40	A	-->	17	O51	PDB	1		2.84

E

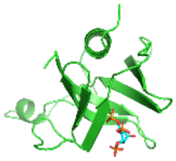

PDB code: rnk1 Ligand PDB

Hydrogen bonds

	ATOM 1						ATOM 2					
	Atom no.	Atom name	Res name	Res no.	Chain		Atom no.	Atom name	Res name	Res no.	Chain	Distance
1.	300	NH1	ARG	18	A	-->	17	O3P	PDB	1		2.81
2.	300	NH1	ARG	18	A	-->	25	O5	PDB	1		3.02
3.	301	NH2	ARG	18	A	-->	17	O3P	PDB	1		3.03
4.	696	OG	SER	40	A	-->	5	O3	PDB	1		2.05
5.	696	OG	SER	40	A	-->	10	O2	PDB	1		2.73
6.	696	OG	SER	40	A	-->	33	O9P	PDB	1		2.73

F

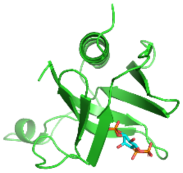

PDB code: rnl2 Ligand PDB

Hydrogen bonds

	ATOM 1						ATOM 2					
	Atom no.	Atom name	Res name	Res no.	Chain		Atom no.	Atom name	Res name	Res no.	Chain	Distance
1.	144	NZ	LYS	9	A	-->	5	O4	PDB	1		2.67
2.	300	NH1	ARG	18	A	-->	1	O41	PDB	1		2.83
3.	301	NH2	ARG	18	A	-->	1	O41	PDB	1		2.52
4.	696	OG	SER	40	A	-->	18	O1	PDB	1		2.00
5.	1115	OH	TYR	67	A	-->	25	O6	PDB	1		2.92

G

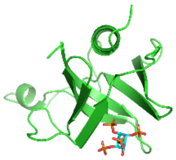

PDB code: rnv2 Ligand PDB

Hydrogen bonds

	ATOM 1						ATOM 2					
	Atom no.	Atom name	Res name	Res no.	Chain		Atom no.	Atom name	Res name	Res no.	Chain	Distance
1.	144	NZ	LYS	9	A	-->	5	O4	PDB	1		2.75
2.	144	NZ	LYS	9	A	-->	28	O6	PDB	1		2.83
3.	300	NH1	ARG	18	A	-->	25	OP3	PDB	1		2.48
4.	674	NE	ARG	39	A	-->	24	OP2	PDB	1		2.93
5.	696	OG	SER	40	A	-->	12	O10	PDB	1		2.52
6.	1115	OH	TYR	67	A	-->	13	O12	PDB	1		2.64

H

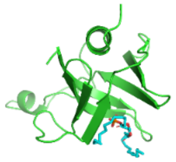

PDB code: szu1   Ligand PDB

Hydrogen bonds

	Atom 1: Atom no.	Atom name	Res name	Res no.	Chain		Atom 2: Atom no.	Atom name	Res name	Res no.	Chain	Distance
1.	300	NH1	ARG	18	A	-->	54	O12	PDB	1		2.85
2.	696	OG	SER	40	A	-->	56	O13	PDB	1		2.15
3.	696	OG	SER	40	A	-->	62	O32	PDB	1		3.13
4.	1115	OH	TYR	67	A	-->	60	O31	PDB	1		2.17

Cre12.g548900

A

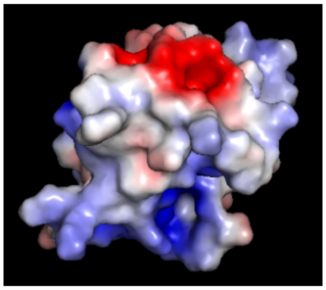

B

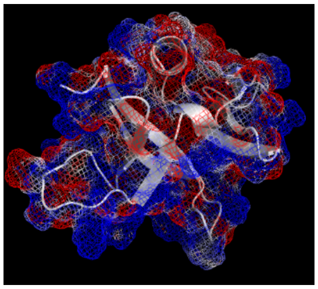

C

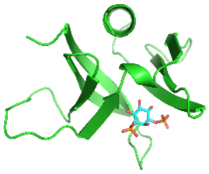

PDB code: rnk9   Ligand PDB

Hydrogen bonds

	Atom 1: Atom no.	Atom name	Res name	Res no.	Chain		Atom 2: Atom no.	Atom name	Res name	Res no.	Chain	Distance
1.	545	NH1	ARG	32	A	-->	5	O1	PDB	1	A	2.74
2.	1235	N	HIS	77	A	-->	21	O4	PDB	1	A	2.98

D

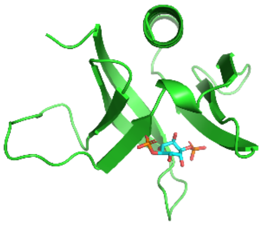

PDB code: rno7   Ligand PDB

Hydrogen bonds

	Atom 1: Atom no.	Atom name	Res name	Res no.	Chain		Atom 2: Atom no.	Atom name	Res name	Res no.	Chain	Distance
1.	148	OG	SER	10	A	-->	10	O5	PDB	1	A	2.91
2.	150	N	GLN	11	A	-->	22	O13	PDB	1	A	3.21
3.	545	NH1	ARG	32	A	-->	10	O5	PDB	1	A	2.92
4.	1235	N	HIS	77	A	-->	18	O1	PDB	1	A	3.28
5.	1270	OH	TYR	78	A	-->	1	O41	PDB	1	A	2.83

E

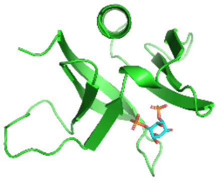

PDB code: rnq8   Ligand PDB

Hydrogen bonds

	Atom 1: Atom no.	Atom name	Res name	Res no.	Chain		Atom 2: Atom no.	Atom name	Res name	Res no.	Chain	Distance
1.	148	OG	SER	10	A	-->	17	O51	PDB	1	A	2.98

F

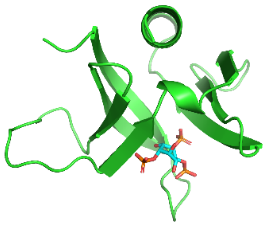

PDB code: rns6 Ligand PDB

Hydrogen bonds

	ATOM 1						ATOM 2					
	Atom no.	Atom name	Res name	Res no.	Chain		Atom no.	Atom name	Res name	Res no.	Chain	Distance
1.	150	N	GLN	11	A	-->	4	O5P	PDB	1	A	2.61
2.	239	NZ	LYS	16	A	-->	32	O7P	PDB	1	A	2.71
3.	545	NH1	ARG	32	A	-->	10	O2	PDB	1	A	2.85
4.	779	N	VAL	47	A	-->	16	O2P	PDB	1	A	2.89
5.	1235	N	HIS	77	A	-->	1	O4P	PDB	1	A	2.98

G

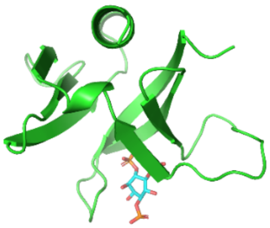

PDB code: rnu9 Ligand PDB

Hydrogen bonds

	ATOM 1						ATOM 2					
	Atom no.	Atom name	Res name	Res no.	Chain		Atom no.	Atom name	Res name	Res no.	Chain	Distance
1.	148	OG	SER	10	A	-->	3	O43	PDB	1	A	2.28
2.	174	N	ASP	13	A	-->	10	O3	PDB	1	A	2.84
3.	334	NE	ARG	21	A	-->	5	O4	PDB	1	A	2.73
4.	334	NE	ARG	21	A	-->	32	O52	PDB	1	A	2.44
5.	543	NE	ARG	32	A	-->	29	O5	PDB	1	A	2.90
6.	545	NH1	ARG	32	A	-->	3	O43	PDB	1	A	2.63

H

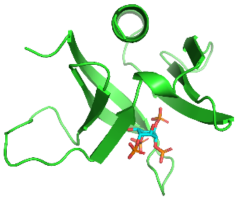

PDB code: ror6 Ligand PDB

Hydrogen bonds

	ATOM 1						ATOM 2					
	Atom no.	Atom name	Res name	Res no.	Chain		Atom no.	Atom name	Res name	Res no.	Chain	Distance
1.	148	OG	SER	10	A	-->	14	O11	PDB	1	A	2.31
2.	150	N	GLN	11	A	-->	3	OP5	PDB	1	A	3.19
3.	779	N	VAL	47	A	-->	24	OP2	PDB	1	A	2.60
4.	1270	OH	TYR	78	A	-->	28	O6	PDB	1	A	2.90

I

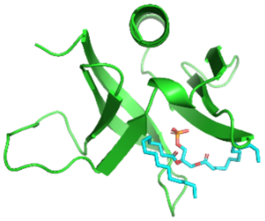

PDB code: pvp3 Ligand PDB

Hydrogen bonds

	ATOM 1						ATOM 2					
	Atom no.	Atom name	Res name	Res no.	Chain		Atom no.	Atom name	Res name	Res no.	Chain	Distance
1.	150	N	GLN	11	A	-->	55	O14	PDB	1	A	2.48
2.	1235	N	HIS	77	A	-->	56	O13	PDB	1	A	2.18

Cre14.g614350

A

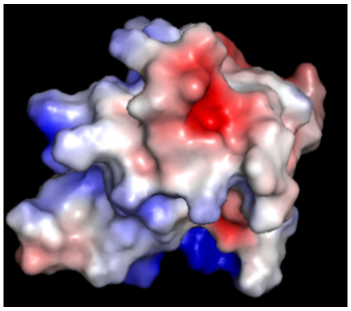

B

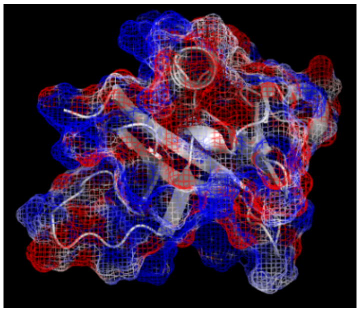

C

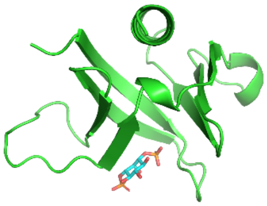

PDB code: qvp6   Ligand PDB

Hydrogen bonds

	ATOM 1						ATOM 2					
	Atom no.	Atom name	Res name	Res no.	Chain		Atom no.	Atom name	Res name	Res no.	Chain	Distance
1.	163	NZ	LYS	10	A	-->	21	O4	PDB	1	A	3.23
2.	321	NH1	ARG	19	A	-->	4	OP2	PDB	1	A	2.56
3.	321	NH1	ARG	19	A	-->	10	O2	PDB	1	A	2.77
4.	372	NH2	ARG	21	A	-->	4	OP2	PDB	1	A	2.39

D

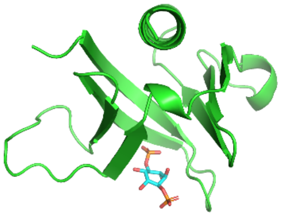

PDB code: qvp7   Ligand PDB

Hydrogen bonds

	ATOM 1						ATOM 2					
	Atom no.	Atom name	Res name	Res no.	Chain		Atom no.	Atom name	Res name	Res no.	Chain	Distance
1.	163	NZ	LYS	10	A	-->	14	O6	PDB	1	A	2.67
2.	191	N	GLU	13	A	-->	22	O13	PDB	1	A	3.23
3.	576	NE1	TRP	32	A	-->	29	O3	PDB	1	A	2.30

E

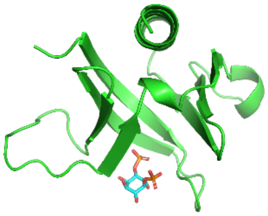

PDB code: qvp9   Ligand PDB

Hydrogen bonds

	ATOM 1						ATOM 2					
	Atom no.	Atom name	Res name	Res no.	Chain		Atom no.	Atom name	Res name	Res no.	Chain	Distance
1.	163	NZ	LYS	10	A	-->	18	O52	PDB	1	A	3.03
2.	321	NH1	ARG	19	A	-->	10	O6	PDB	1	A	2.49
3.	367	NE	ARG	21	A	-->	29	O2	PDB	1	A	2.66
4.	372	NH2	ARG	21	A	-->	29	O2	PDB	1	A	2.43

F

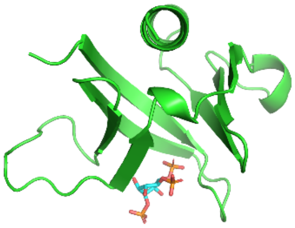

PDB code: qvq1   Ligand PDB

Hydrogen bonds

	ATOM 1						ATOM 2					
	Atom no.	Atom name	Res name	Res no.	Chain		Atom no.	Atom name	Res name	Res no.	Chain	Distance
1.	321	NH1	ARG	19	A	-->	10	O2	PDB	1	A	2.19
2.	321	NH1	ARG	19	A	-->	18	O1P	PDB	1	A	2.66
3.	367	NE	ARG	21	A	-->	21	O6	PDB	1	A	2.38
4.	779	NH1	ARG	45	A	-->	16	O2P	PDB	1	A	3.18

G

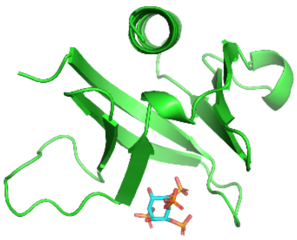

PDB code: pvq2   Ligand PDB

Hydrogen bonds

	ATOM 1						ATOM 2					
	Atom no.	Atom name	Res name	Res no.	Chain		Atom no.	Atom name	Res name	Res no.	Chain	Distance
1.	163	NZ	LYS	10	A	-->	3	OP5	PDB	1	A	3.04
2.	321	NH1	ARG	19	A	-->	32	O5	PDB	1	A	2.87
3.	321	NH1	ARG	19	A	-->	35	OP7	PDB	1	A	2.62
4.	367	NE	ARG	21	A	-->	17	O2	PDB	1	A	2.36
5.	372	NH2	ARG	21	A	-->	23	OP1	PDB	1	A	2.08

H

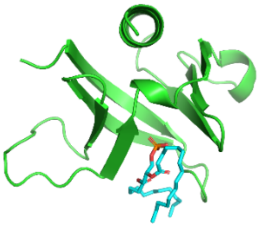

PDB code: rcp2 Ligand PDB

Hydrogen bonds

	<----- A T O M 1 ----->						<----- A T O M 2 ----->					
	Atom no.	Atom name	Res name	Res no.	Chain		Atom no.	Atom name	Res name	Res no.	Chain	Distance
1.	163	NZ	LYS	10	A	-->	56	O13	PDB	1	A	2.64

Cre14.g616050

A

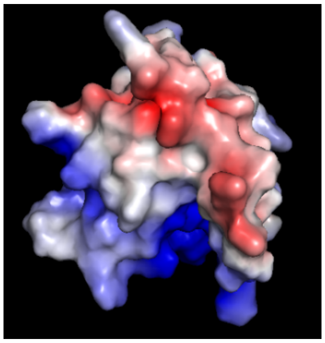

B

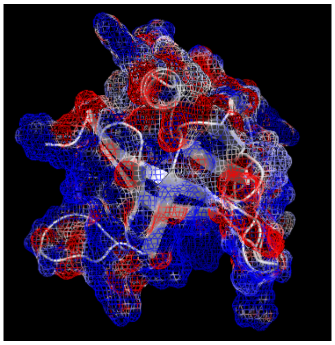

C

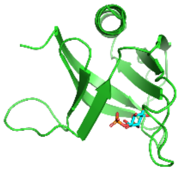

PDB code: qvq4 Ligand PDB

Hydrogen bonds

	<----- A T O M 1 ----->						<----- A T O M 2 ----->					
	Atom no.	Atom name	Res name	Res no.	Chain		Atom no.	Atom name	Res name	Res no.	Chain	Distance
1.	1268	OG	SER	83	A	-->	21	O4	PDB	1		3.12
2.	1281	NE	ARG	84	A	-->	29	O6	PDB	1		2.55

D

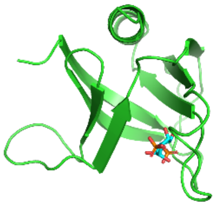

PDB code: qwj2 Ligand PDB

Hydrogen bonds

	<----- A T O M 1 ----->						<----- A T O M 2 ----->					
	Atom no.	Atom name	Res name	Res no.	Chain		Atom no.	Atom name	Res name	Res no.	Chain	Distance
1.	143	NZ	LYS	10	A	-->	4	O42	PDB	1		2.91
2.	143	NZ	LYS	10	A	-->	5	O4	PDB	1		2.96
3.	1268	OG	SER	83	A	-->	18	O1	PDB	1		2.26
4.	1274	N	ARG	84	A	-->	14	O6	PDB	1		3.06
5.	1281	NE	ARG	84	A	-->	29	O3	PDB	1		3.17

E

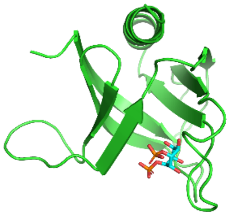

PDB code: qxm8 Ligand PDB

Hydrogen bonds

	<----- A T O M 1 ----->						<----- A T O M 2 ----->					
	Atom no.	Atom name	Res name	Res no.	Chain		Atom no.	Atom name	Res name	Res no.	Chain	Distance
1.	536	NE1	TRP	32	A	-->	17	O51	PDB	1		2.66
2.	1268	OG	SER	83	A	-->	3	O13	PDB	1		2.98
3.	1274	N	ARG	84	A	-->	10	O6	PDB	1		2.99

F

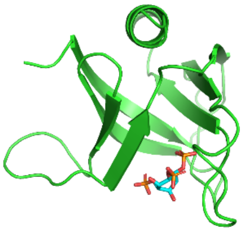

PDB code: qxn5   Ligand PDB

Hydrogen bonds

	ATOM 1						ATOM 2					
	Atom no.	Atom name	Res name	Res no.	Chain		Atom no.	Atom name	Res name	Res no.	Chain	Distance
1.	143	NZ	LYS	10	A	-->	17	O3P	PDB	1		3.17
2.	143	NZ	LYS	10	A	-->	25	O5	PDB	1		2.62
3.	143	NZ	LYS	10	A	-->	31	O8P	PDB	1		2.90
4.	696	NH2	ARG	43	A	-->	16	O2P	PDB	1		2.93
5.	1268	OG	SER	83	A	-->	1	O4P	PDB	1		2.68
6.	1281	NE	ARG	84	A	-->	32	O7P	PDB	1		2.87
7.	1284	NH2	ARG	84	A	-->	32	O7P	PDB	1		2.97

G

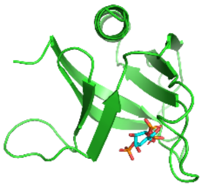

PDB code: qxn8   Ligand PDB

Hydrogen bonds

	ATOM 1						ATOM 2					
	Atom no.	Atom name	Res name	Res no.	Chain		Atom no.	Atom name	Res name	Res no.	Chain	Distance
1.	143	NZ	LYS	10	A	-->	4	O42	PDB	1		3.02
2.	143	NZ	LYS	10	A	-->	32	O52	PDB	1		2.85

H

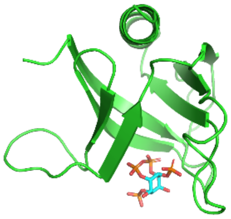

PDB code: rps3   Ligand PDB

Hydrogen bonds

	ATOM 1						ATOM 2					
	Atom no.	Atom name	Res name	Res no.	Chain		Atom no.	Atom name	Res name	Res no.	Chain	Distance
1.	143	NZ	LYS	10	A	-->	1	OP4	PDB	1		2.45
2.	143	NZ	LYS	10	A	-->	13	O12	PDB	1		2.25
3.	339	NE	ARG	21	A	-->	12	O10	PDB	1		3.08
4.	696	NH2	ARG	43	A	-->	17	O2	PDB	1		2.50
5.	696	NH2	ARG	43	A	-->	21	O1	PDB	1		2.68

I

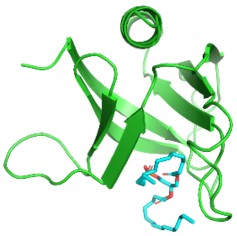

PDB code: pyz3   Ligand PDB

Hydrogen bonds

	ATOM 1						ATOM 2					
	Atom no.	Atom name	Res name	Res no.	Chain		Atom no.	Atom name	Res name	Res no.	Chain	Distance
1.	143	NZ	LYS	10	A	-->	56	O13	PDB	1		2.53
2.	339	NE	ARG	21	A	-->	54	O12	PDB	1		2.95
3.	342	NH2	ARG	21	A	-->	54	O12	PDB	1		2.93
4.	693	NE	ARG	43	A	-->	54	O12	PDB	1		3.11
5.	696	NH2	ARG	43	A	-->	45	O22	PDB	1		3.33

Cre16.g653700

A

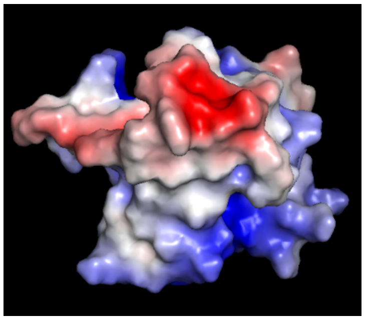

B

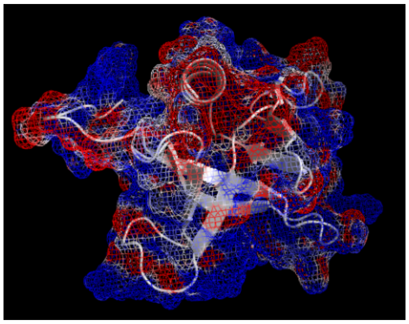

C

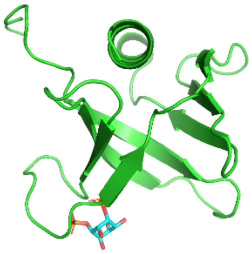

PDB code: rbx8   Ligand PDB

Hydrogen bonds

	<----- A T O M 1 ----->						<----- A T O M 2 ----->					
	Atom no.	Atom name	Res name	Res no.	Chain		Atom no.	Atom name	Res name	Res no.	Chain	Distance
1.	232	NE	ARG	26	A	-->	29	O2	PDB	1		2.91
2.	235	NH2	ARG	26	A	-->	29	O2	PDB	1		2.52
3.	345	N	LYS	41	A	-->	1	O11	PDB	1		2.90
4.	345	N	LYS	41	A	-->	17	O51	PDB	1		2.94
5.	423	NE1	TRP	50	A	-->	10	O6	PDB	1		2.39

D

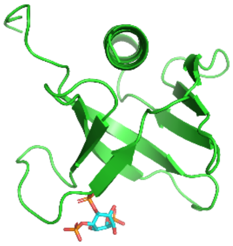

Hydrogen bonds

	<----- A T O M 1 ----->						<----- A T O M 2 ----->					
	Atom no.	Atom name	Res name	Res no.	Chain		Atom no.	Atom name	Res name	Res no.	Chain	Distance
1.	232	NE	ARG	26	A	-->	25	O5	PDB	1		2.74
2.	232	NE	ARG	26	A	-->	31	O8P	PDB	1		2.54
3.	234	NH1	ARG	26	A	-->	17	O3P	PDB	1		2.66
4.	235	NH2	ARG	26	A	-->	17	O3P	PDB	1		2.29
5.	235	NH2	ARG	26	A	-->	25	O5	PDB	1		2.23
6.	345	N	LYS	41	A	-->	33	O9P	PDB	1		3.05
7.	353	NZ	LYS	41	A	-->	10	O2	PDB	1		2.79
8.	399	N	LYS	48	A	-->	4	O5P	PDB	1		3.24

E

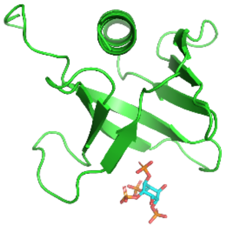

Hydrogen bonds

	<----- A T O M 1 ----->						<----- A T O M 2 ----->					
	Atom no.	Atom name	Res name	Res no.	Chain		Atom no.	Atom name	Res name	Res no.	Chain	Distance
1.	235	NH2	ARG	26	A	-->	13	O12	PDB	1		2.53
2.	235	NH2	ARG	26	A	-->	36	OP8	PDB	1		2.38

F

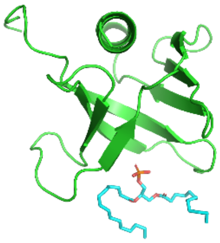

Hydrogen bonds

	<----- A T O M 1 ----->						<----- A T O M 2 ----->					
	Atom no.	Atom name	Res name	Res no.	Chain		Atom no.	Atom name	Res name	Res no.	Chain	Distance
1.	215	NE2	HIS	24	A	-->	60	O31	PDB	1		2.93

Figure S1. Surface electrostatics and binding profiles of the top PH domain models. **A.** Surface electrostatic potentials of PH domain color-graded from -4 kT/e (red) to +4 kT/e (blue). **B.** Mesh presentation electrostatic potential, color-graded from -4 kT/e (red) to +4 kT/e (blue). **C-F.** Binding scenarios with phosphoinositides.
